# Sociosexual Exposure Has Opposing Effects on Male and Female Actuarial Senescence in the Fruit Fly *Drosophila melanogaster*

**DOI:** 10.1093/gerona/glad215

**Published:** 2023-09-11

**Authors:** Wayne G Rostant, Janet S Mason, Nicholas West, Alexei A Maklakov, Tracey Chapman

**Affiliations:** School of Biological Sciences, University of East Anglia, Norwich, UK; School of Biological Sciences, University of East Anglia, Norwich, UK; School of Biological Sciences, University of East Anglia, Norwich, UK; School of Biological Sciences, University of East Anglia, Norwich, UK; School of Biological Sciences, University of East Anglia, Norwich, UK; (Biological Sciences Section)

**Keywords:** Lifespan, Mortality, Reproductive costs

## Abstract

Males and females rarely express the same length of life. Here, we studied how sociosexual exposure shapes male and female age-specific mortality rates in *Drosophila melanogaster*. We maintained focal females and males within large, replicated cohorts throughout life with individuals of the same or opposite sex. Consistent with previous works, we found that females kept throughout their lives with males had only half the lifespan of those maintained throughout life at the same density in same-sex cohorts. In contrast, only a small lifespan decrease was observed in the corresponding male treatments and the reduction in male lifespan following exposure throughout life to other males or females was similar. Deconvolution of underlying aging parameters revealed that changes in lifespan were underpinned by opposing effects on actuarial aging in males versus females. Exposure to the opposite or same sex increased initial mortality rate in both sexes. However, in females, increasing exposure to males increased the rate of aging, while increasing exposure to females actually decreased it. The effects were in the opposite direction in males and were much smaller in magnitude. Overall, the findings were consistent with reports suggesting that exposure to the same versus opposite sex can affect survival differently in males and females. However, they also reveal a new insight—that overall lifespan can be underpinned by key differences in actuarial senescence in each sex. The findings suggest that responses to same or opposite sex exposure may have fundamentally and qualitatively different physiological consequences for health in males and females.

## Sex Differences in Lifespan

In organisms with separate sexes, male and female lifespans are often characteristically different. Sex differences in lifespan (SDL) can vary in direction and magnitude across species (1–4) and can also show plasticity within species in response to variation in diet and reproductive activity (5–12). However, much about the determination of sex-specific lifespan remains unclear (3,13). One leading explanation for variation in lifespan in males and females within and across species centers on sexual selection, in which SDL occurs because each sex is selected to increase its own fitness by adopting sex-specific life histories that can be manifested as differing trade-offs (eg, between reproduction and lifespan) and thus costs of reproduction (14–20). The sex-specific selection hypothesis was developed from observations of the association between length of life in males and females with different mating systems (8,21–23). The underlying idea is that, as a consequence of anisogamy, males and females have different routes to maximize their fitness, resulting in selection for sex-specific life histories (2,16,24,25). This may necessarily result in different lengths of life because the optimal resolutions of life-history trade-offs differ between the sexes (26,27). A lifespan that is the same for both sexes cannot resolve these conflicting strategies. An extension of this idea is that via SDL, each sex can adopt a different, fitness-maximizing life history and hence also reduce sexual conflict (14,15,28,29). Indeed, a study in which sex-specific selection was experimentally imposed showed that SDL can either widen or evolve into sexual monomorphism in lifespan in just 20 generations (17). Selection on age at reproduction can also result in the adaptive evolution in one sex affecting senescence in the other (30). If such sexual conflict effects are common and large in magnitude it would suggest that the portion of sex-specific lifespan they influence could be missed in experiments conducted on individuals maintained throughout all or most of their lives on their own or in single sex environments.

That exposure to the same or other sex can affect lifespan in males and females is well known (14,15,28,29,31). Sex-specific survival costs of reproduction can arise due to variation in reproductive investment (in eggs and ejaculates), direct interactions or indirect perception of the same or other sex (32–37). Experimental studies in *Drosophila melanogaster* have delineated these different effects (38–42) showing that female lifespan is reduced by elevated mating, receipt of seminal fluid proteins from males during mating (43–49) elevated egg production (50,51), and physical courtship interactions (46). Female survival costs arising from mating appear to outweigh those associated with the production of eggs per se (52,53). Whether such survival costs of mating in female parents are balanced by potential fitness benefits to their offspring is not clear, with evidence both for (42) and against (54) this possibility. Similar studies on males show minimal impacts on survival of mating (55), and suggest instead that energetic costs of courtship activity are significant (56) though surprisingly limited in extent (57). That such costs predominate for males is also suggested by the finding that male survival is decreased significantly by exposure to other males (31,58,59). Physiological survival costs of male reproductive system activation per se are suggested by the finding that serial lengthy matings in *D melanogaster* can decrease male survival and late-life fertility (60). There is also increasing interest in sex-specific effects of same sex exposure on lifespan, particularly given recent findings that exposure of older to younger individuals in *D melanogaster* can extend lifespan of both sexes (61). It has been shown experimentally that the survival of males can be reduced to a greater degree by exposure to same sex individuals than is true for corresponding groups of females in different insects (31). However, the effects on age-specific mortality rates have not yet been investigated in this context. Exactly how the potentially differing impacts of same and opposite sex exposure affect male and female lifespan via different mortality rates is the new topic of investigation here.

Direct comparisons of survival in each sex under varying sociosexual exposures are often hampered by the use of varying experimental frameworks across studies. Only common set-ups can allow the magnitude of lifespan effects to be estimated and to avoid potential confounds arising from sex-specific responses to diet and other environmental factors. The potential for confusion is exemplified in studies of *D melanogaster.* Sex differences in lifespan are widely reported (62–64) but are not always observed (65,66). Variation in which sex lives longest is also rife and affected by several key factors. One is the sensitivity of female lifespan in particular to mating status (45,48,64,67). For example, single matings at the start of life have minimal effects on female lifespan (4,46), yet intermittent or continual exposure to males can shorten it dramatically (45,48). Female lifespan also shows heightened sensitivity to dietary variation (6,18,68). There are variable and apparently sex-specific effects of social groupings on male and female lifespan (31,64). That there are likely to be differing magnitudes of effects of same versus opposite sex exposure on lifespan in males and females is indicated by these and many other studies. However, as such effects are rarely tested for within the same study or experimental set-up, the strength of the selective forces shaping sex-specific lifespan remains unknown. One example in which a common experimental set-up was used for both sexes is the study of Iliadi et al. (64) in *D melanogaster.* This showed that lifespan differences between 2 strains, and which sex lived longer overall, was altered by social exposure (virgin, single sex, or mixed-sex groupings). Strain differences in survival were also low in flies held in mixed-sex treatments (64) consistent with previous findings (69,70). Even more rare are studies like these of both sexes within the same framework that also include measures of actuarial aging (30,64,71,72). These are highly useful as they can document the trajectory of aging (when it starts and how fast it proceeds) and are crucial to understanding the mechanisms of sex differences in aging (64). Their omission is likely due to the large sample sizes required to reliably estimate underlying aging parameters (73).

Our main aim was to address the knowledge gaps identified above by testing the key hypothesis that there are qualitatively and quantitatively different effects of same versus opposite sex exposure on survival in males and females (16,43). We did this by measuring the effects of sociosexual exposure in each sex in the same experimental set-up and with sufficient power to permit the derivation of parameters of actuarial aging.

## Method

### Experimental Rearing Conditions

Experiments were conducted in a 25°C humidified room held under a 12-hour light:12-hour dark cycle. Wild-type Dahomey flies were used throughout. This wild-type stock has been maintained at large population sizes in overlapping generations in cage culture since the 1970s on sugar-yeast-agar (SYA) medium (100 g Brewer’s yeast, 50 g sucrose, 15 g agar, 30 mL Nipagin (10% solution), 3 mL propionic acid, and 0.97 L water per liter of medium), which was used throughout. Flies were sampled from the population cages by allowing females to oviposit on agar-grape juice plates (50 g agar, 600 mL red grape juice, 42 mL Nipagin (10% solution), 1.1 L water). Larvae were collected from these plates and reared under a controlled density of 100 per vial. All adults were collected and separated by sex within 8 hour of eclosion and stored in groups of 20 in vials (75 × 25 mm). To enable identification of focal versus nonfocal individuals, nonfocal flies of each sex were created by clipping the wing tips of 1-day-old adults under CO_2_ anesthesia.

### Set-up of Same and Opposite Sex Social Exposure Treatments

We set up 5 replicate cages (192 × 128 × 10.6 mm) for each of 4 social treatments for each sex (5 × 4 × 2 = 40 cages in total). The 4 social exposure treatments were:

(i) Focals alone: Focal males or females held throughout life in single sex groups (*n* = 200 focal individuals per cage × 5 replicate cages × 2 sexes). Cage density = 200 focals per replicate cage.
(ii) Intermittent (opposite sex) exposure: Focal males or females intermittently exposed throughout life to young individuals of the opposite sex (*n* = 200 focals + 200 nonfocals of the opposite sex present for 1 day out of every 4 × 5 replicate cages × 2 sexes). Cage density = 200 focals + 200 nonfocals (400 flies) per replicate cage.
(iii) Continuous (opposite sex) exposure: Focal males or females continuously exposed throughout life to young individuals of the opposite sex (*n* = 200 focals + 200 nonfocals of the opposite sex × 5 replicate cages × 2 sexes). Cage density = 200 focals + 200 nonfocals (400 flies) per replicate cage.
(iv) Continuous (same sex) exposure: Focal males or females continuously exposed throughout life to young individuals of the same sex (*n* = 200 focals and 200 nonfocals of the same sex; 5 replicate cages × 2 sexes). Cage density = 200 focals + 200 nonfocals (400 flies) per replicate cage.

Each cage was supplied with food through the side with 3 vials (75 high × 25 mm diameter), each containing 7 mL SYA medium. Food vials were renewed twice every 4 days in a repeating 1 + 3 day cycle. To keep levels of activity high, all nonfocal individuals of each sex in treatments (ii–iv) were replaced with new young 3–7-day-old nonfocal individuals every 4 days by using CO_2_ anesthesia, with completely new sets of nonfocals being introduced every second 4-day cycle. We always controlled for CO_2_ exposure by similarly anesthetizing with CO_2_ all cages (including the focal alone treatments) to the same extent regardless of whether there were any nonfocal flies to remove or replace. Note that the density of the focals alone treatment (i) was initially half that of the continuous social treatments (iii and iv) and that the density of the intermittent treatment varied periodically between the two, depending upon the phase of the experiment. Higher densities could have a negative effect on lifespan and thus potentially decrease our ability to detect lifespan differences between treatments (61,74) if they were present. However, we also note that even the highest initial densities of flies can be considered low because of the large cages used. Comparisons between the continuous treatments ((iii) and (iv) above) are also controlled for density. Thus, any variation in density across treatments was expected to have minimal confounding effects on survival in this experimental set-up. Lifespan of focal males and females was recorded daily until all focal individuals were dead. Dead individuals were removed from the cages every 1–2 days by aspiration (inserting an aspiration tube into the cage through a side opening). For mixed-sex treatments the sex ratio was held at 1:1, by removing using aspiration a nonfocal individual if a focal individual had died.

We set up the experiments for each sex separately under exactly the same conditions, in series. Female and male experiments were conducted separately due to logistical constraints and the need to generate the large sample sizes required. Five replicate cages of 200 focal individuals of each sex and 4 social treatments were set up (5 × 200 × 4 = 4 000 females and 4 000 males set up and scored in total).

### Statistical Analysis

**Figure 1.**
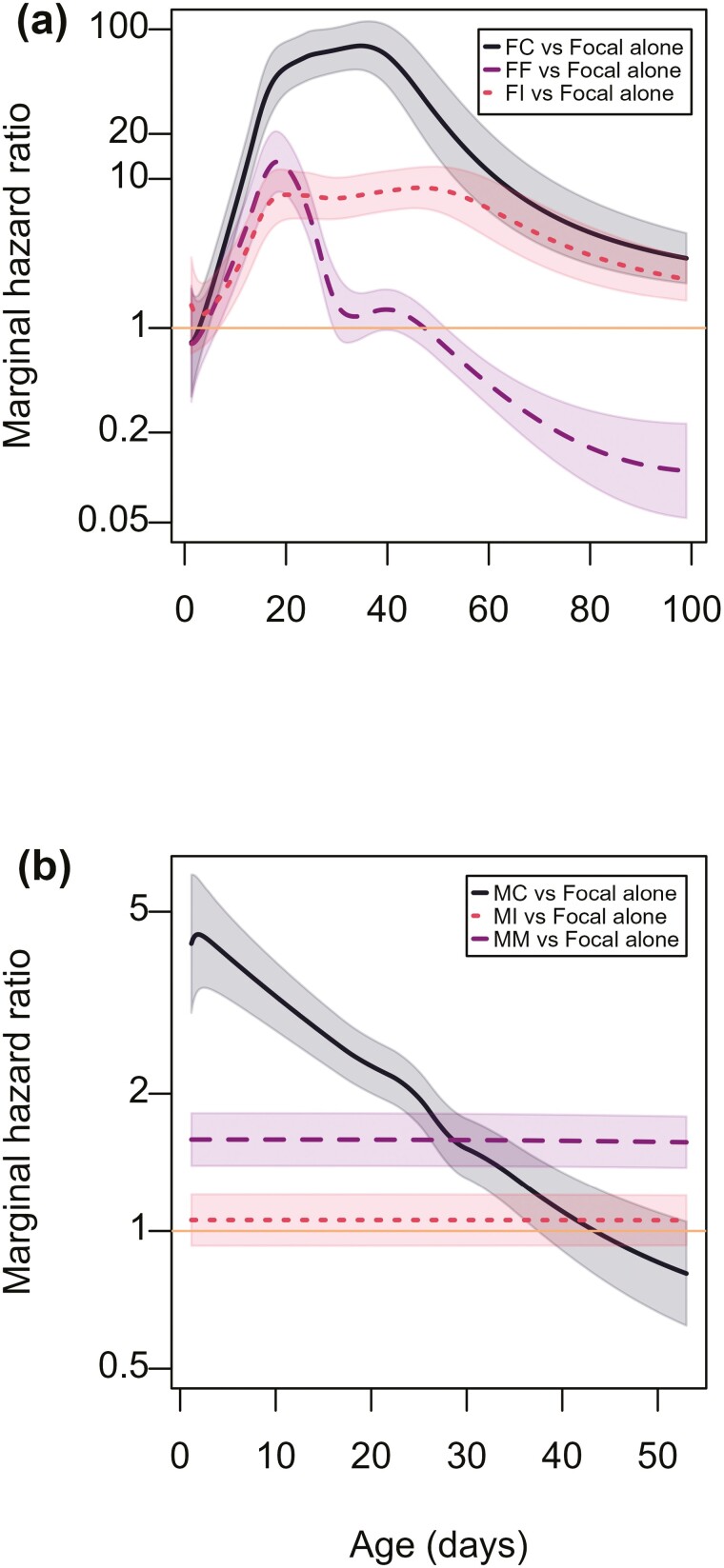
Marginal hazard ratios, for each same or opposite sex exposure treatment relative to the baseline female (F) or male (M) focal alone groups, against age in days. Shown are the plots of the best-fit generalized survival models (GSM) (with 95% confidence intervals) for (A) Females and (B) Males. Treatments are Focal alone (females or males kept in single sex groups throughout life); FC/MC (females or males exposed continuously to young nonfocals of the opposite sex throughout life); FI/MI (females or males exposed intermittently to young nonfocals of the opposite sex throughout life); FF/MM (females or males exposed continuously to young nonfocals of the same sex throughout life). Hazard ratios were all nonproportional (not flat) in the best-fit female model. Thus, in females, the hazard ratios varied significantly across the lifetime and in different ways in the different exposure treatments. For males, the pattern was different, with the hazard ratio of MC treatment males continuously exposed to females being the only one that was nonproportional through time.

Analyses for focal females and males proceeded separately. Due to nonindependence for individual-level data (cage effect) and nonproportionality in hazards across treatments, generalized survival models (GSMs) (75) were fitted to age-specific individual mortality to allow both the inclusion of the additive random effect of replicate population (by including a frailty term) and time dependence in the hazard ratios. Generalized survival models were implemented using the stpm2() function in the R package rstpm2 (76). We determined the appropriate time dependence for both the baseline hazard (for the focal sex alone treatment) and the treatment coefficients by fitting models with different degrees of freedom on the relevant natural cubic spline terms and comparing them using the Akaike Information Criterion (AIC). Given the nonproportionality of hazards (Figure 1), 2 further approaches were used to complement the GSM fitting. First, average Cox proportional hazard ratios were determined using the coxphw() function in the coxphw package (77). Second, we employed Restricted Mean Survival Time (RMST) as an alternative to the hazard ratio (78). Modeled mean individual survival for each treatment was derived using GSM-predicted Restricted Mean Survival Times (RMST_GSM_). Further analysis of mean survival at the population level was facilitated by using the rmst() function in the RISCA package (79) to calculate nonparametric RMST via the trapezoidal rule (area under the Kaplan-Meier survival curves), hereafter RMST_K-M_, and subjecting the outputs to simple ANOVA using aov() in the car package (80).

Actuarial aging was investigated at the population level by fitting age-specific mortality data for each focal sex within each population to a series of 2-parameter parametric aging models (Supplementary Table 1). Comparisons using AIC showed that overall, the Gompertz mortality function was the best fit to the female data set and, for consistency, was applied to both female and male populations for subsequent description and analysis. Under this model mortality is fitted as:


$$\begin{array}{l} \mu \left(x\right)=\alpha {e^{\beta }}^{x} \end{array}$$

where *x* is age (days), µ(*x*) is age-specific mortality, *α* is background mortality, and *β* is rate of mortality increase per day. As aging parameters were negatively correlated, we modeled their response to treatments using a multivariate framework as follows. We used a nonparametric (permutational) MANOVA (81) as implemented by the adonis2() function in the “vegan” package (82). Order-of-magnitude differences in mean and variance between *α* and *β* parameters were minimized by scaling each response prior to analysis and Euclidean pairwise distances were calculated prior to permutation significance tests with pseudo-*F* ratios.

For both female and male GSMs, there was strong evidence for nonproportionality in the hazard ratios across treatments (Figure 1) with the best-fit models, according to AIC, having time-varying components. For both female and male GSMs, the baseline hazards (ie, focal sex alone treatment) were flexibly fit with a natural cubic spline basis degree of freedom = 6. Using AIC there was strong evidence for including replicate population as a random effect (using Gamma shared frailties) and nonproportionality in the hazard ratios across treatments (Figure 1) with the best-fit models having time-varying components. In the model for female data, all treatments showed hazard ratios that varied over time in comparison to the baseline (focal female alone) control (Figure 1A). For the male data model, the hazard ratio of the continuous male exposure treatment similarly varied over time in comparison to the baseline (focal male alone) control, whereas the hazard ratios for the intermittent female and continuous female exposure treatments were proportional to the baseline control (Figure 1B). This motivated the subsequent use of weighted proportional hazards as summary statistics to indicate average effects over lifespan (Table 1).

**Table 1.** Summary Statistics for Lifespan and Actuarial Aging

Focal Sex	Treatment	Mean Lifespan: RMST_GSM_ (days ± CI)	Weighted HR cf Focal Alone From coxphw() (±CI)	Gompertz Aging Parameters	
				Initial Mortality (α) ×10^−4^ (± CI)	Rate of Increase of Mortality (β) ×10^−2^ (±CI)
Female	Alone focal ♀	44.0 (41.9–46.0)	—	7.09 (1.94–13.59)	11.31 (9.12–13.43)
	Intermittent (opposite sex) exposure of focal ♀ to ♂	30.3 (28.5–32.1)	4.09 (3.68–4.55)	16.56 (6.17–27.18)	13.30 (11.16–15.73)
	Continuous (opposite sex) exposure of focal ♀ to ♂	19.1 (17.8–20.4)	13.68 (11.95–15.65)	48.86 (26.37–78.65)	16.98 (14.73–19.62)
	Continuous (same sex) exposure of focal ♀ to ♀	36.9 (33.6–40.2)	2.04 (1.81–2.29)	73.81 (46.12–97.00)	4.77 (3.76–6.07)
Male	Alone focal ♂	29.1 (28.2–30.0)	—	42.57 (32.62–51.30)	9.39 (8.77–10.04)
	Intermittent (opposite sex) exposure of focal ♂ to ♀	28.6 (27.7–29.4)	1.09 (0.99–1.21)	48.24 (41.47–54.04)	9.10 (8.60–9.82)
	Continuous (opposite sex) exposure of focal ♂ to ♀	22.0 (21.1–22.9)	2.34 (2.11–2.59)	115.88 (94.07–143.56)	8.01 (7.15–9.00)
	Continuous (same sex) exposure of focal ♂ to ♂	25.1 (24.3–25.8)	1.54 (1.40–1.70)	46.73 (39.11–54.41)	10.96 (9.99–11.75)

*Note*: HR = Hazard ratio; RMST_GSM_ = GSM-predicted Restricted Mean Survival Times.

## Results

### Female Lifespan and Actuarial Aging Patterns

**Figure 2.**
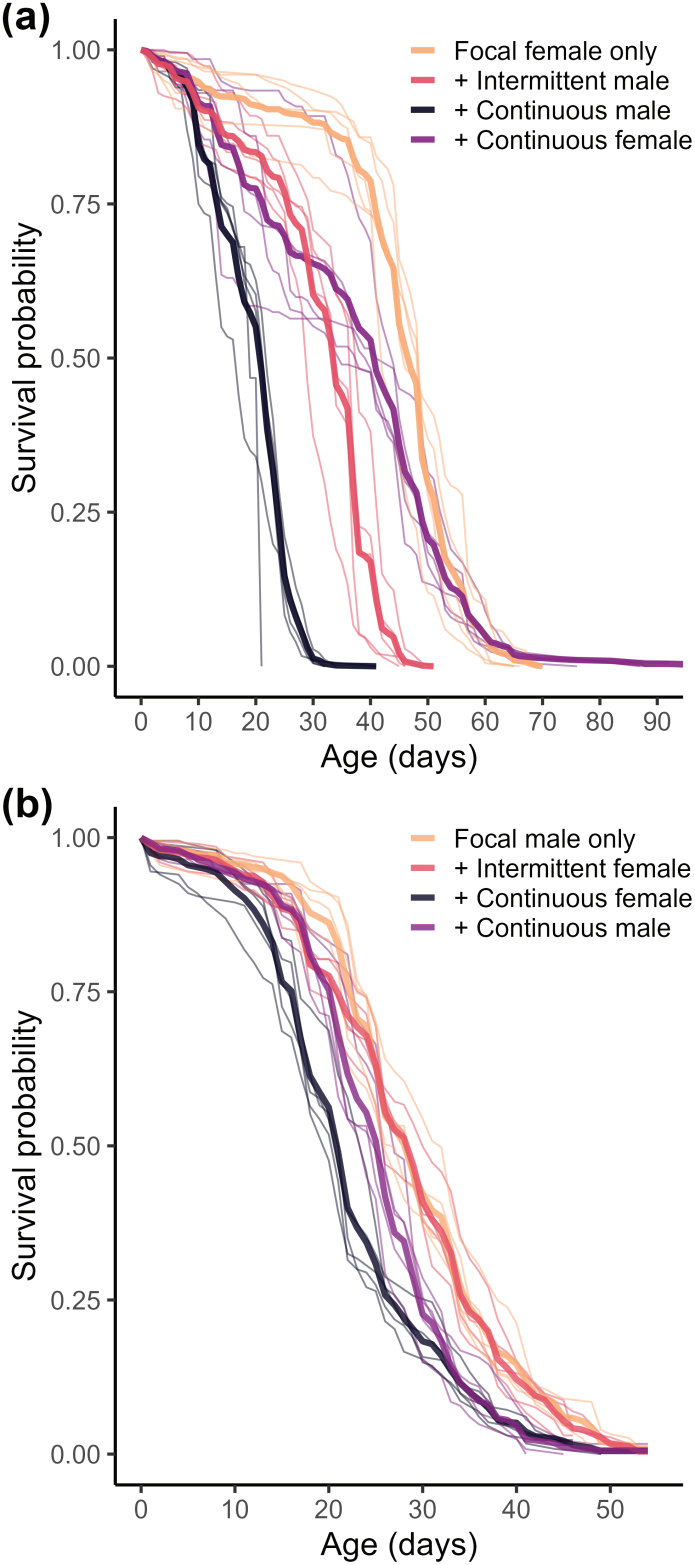
Survival curves for (A) females and (B) males held under different sociosexual exposures. There were 5 replicate populations for each of the following treatment in each of the female and male experiments. Each treatment consisted of 5 replicate populations of *n* = 200 focal individuals. Focal alone = 200 focal flies; Focal + Intermittent = 200 focals + 200 nonfocals of the opposite sex placed in the cage for 1 day in every 4 throughout life; Focal + Continuous = 200 focals + 200 young nonfocals of the opposite or same sex continuously throughout life. Nonfocals were swapped for fresh individuals of the same cohort every 4 days and exchanged for new young nonfocal flies every 8 days i.e. second after every 4-day cycle). Thin lines represent individual replicate cage population trajectories; thick lines average survival trajectory for each treatment.

Focal “alone” female lifespan was longer than for males (RMST_GSM_ females = 44.0 days [bootstrapped 95% CI = 41.9, 46.0]; RMST_GSM_ males = 29.1 days [28.2, 30.0]; Table 1). The lifespan of these females also exhibited a long tail, with the oldest females living in excess of 90 days (Figure 2A). As expected, exposure of focal females continuously to nonfocal males caused a large and significant decrease in survival (RMST_K-M_, ANOVA; *F*_3,16_ = 81.1, *p* < .001; all post hoc contrasts significant after Benjamini–Hochberg correction, *p* < .05). The individual mean lifespan of females continually exposed to males was less than half (RMST_GSM_ = 19.1 days [17.8, 20.4]) that of “alone” focal females (RMST_GSM_ = 44.0 days [41.9, 46.0]). The lifespan of the intermittent (opposite sex) treatment females exposed to males intermittently fell in the middle (RMST_GSM_ = 30.3 days [28.5, 32.1]). Interestingly, females held continuously with nonfocal females also had shorter lifespans (RMST_GSM_ = 36.9 days [33.6, 40.2]) than the baseline alone focal females (post hoc RMST_K-M_ contrast *t* = 4.29, *p* < .001). Part of the reason for the shorter lifespan of the continuous (same sex) females, in comparison to “alone” treatment females could be a density effect—each replicate of the focal alone comprised 200 focals per cage and all other treatments had 200 focals + 200 nonfocals. However, the demographic survival patterns aren’t consistent with this idea—for example, that of the continuous (same sex) treatment females (constant survival loss) was markedly different to that of females from all other treatments that varied across the 2 densities (a survival plateau followed by drop off in survival probability). We also compared directly the effects of males on female survival for the treatments that were initiated at the same densities, ie, continuous (same sex) treatment females in comparison to intermittent (opposite sex) and continuous (opposite sex) treatment females (post hoc RMST_K-M_ [cont. same sex—interm. opposite sex] contrast *t* = 3.62, *p* = .002; and [cont. same sex—interm. opposite sex] contrast *t* = 10.75, *p* < .001, respectively). This shows that the deleterious impact of males on shortening female lifespan occurs over and above any simple initial density effects.

**Figure 3.**
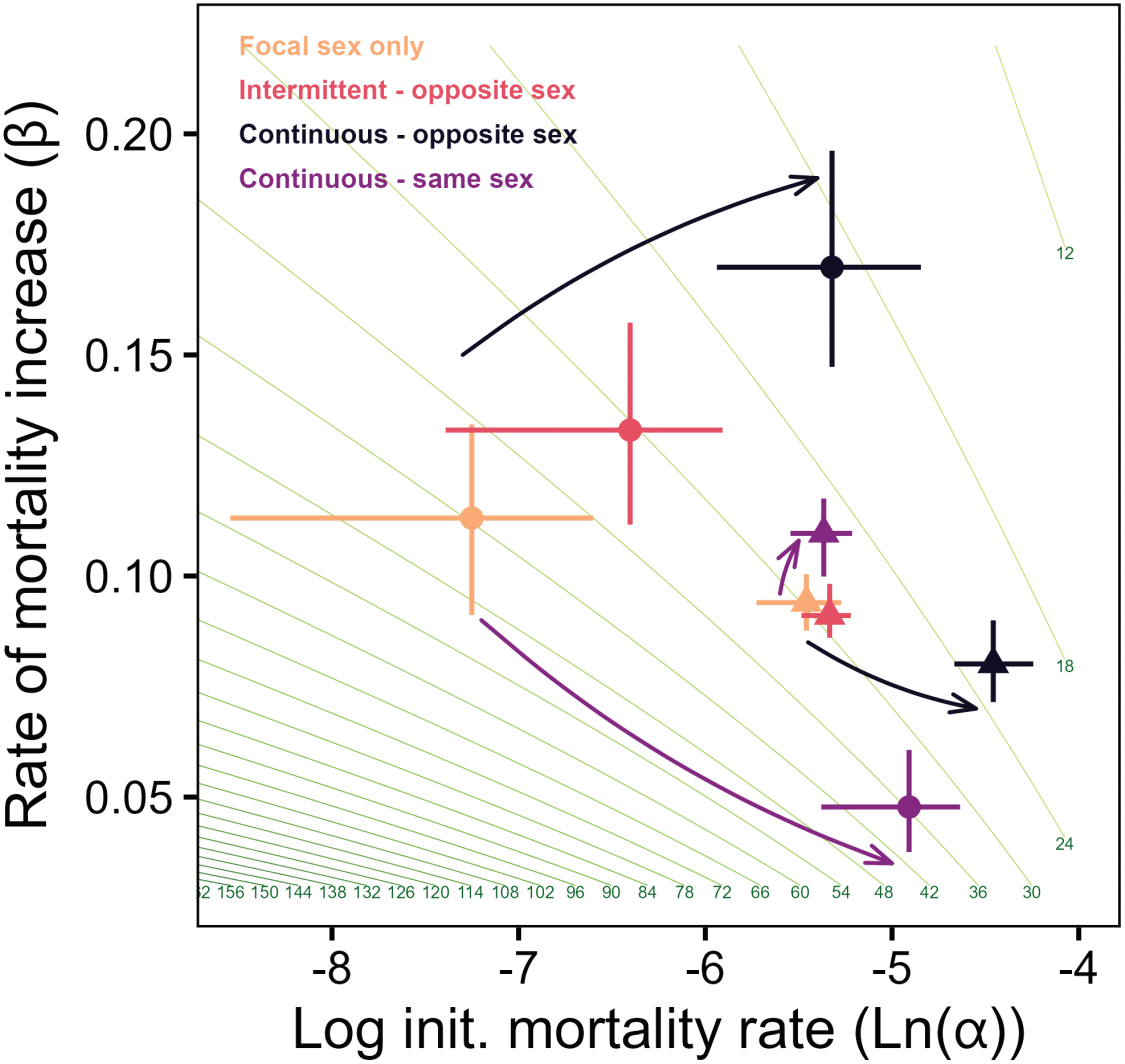
Initial mortality and aging rate parameters for females and males held under different sociosexual exposures. Shown are the fitted Gompertz aging parameters for both females (circles) and males (triangles), with the same or opposite sex exposure treatments indicated. Each point represents the mean fitted Gompertz parameter estimates for the social environmental treatment (± bootstrapped 95% CI). Black arrows indicate the effect of increasing exposure to the opposite sex, while purple arrows indicate effect of increased exposure to same sex (ie, increased density). Green contours show the Gompertz-predicted median lifespan in days for the parameter space (6 days between each contour line). Movement up and to the right results in decreased median lifespan, while to the bottom and left results in increased median lifespan.

Analysis of aging parameters (Figure 3, Table 1) illuminated these patterns further. Female aging parameters (*α, β*) were significantly affected by social environment (permutational MANOVA; *F*_3,16_ = 11.31, *p* < .001; all post hoc contrasts significant after Benjamini–Hochberg correction, *p* < .05). Different aging patterns (arrows in Figure 3) were found depending on the nonfocal sex to which females were exposed, even within treatments set up at the same initial density. With increasing exposure to males, females experienced increases along both axes of aging parameters. Thus, exposure to males increased both the initial (background) rate of mortality (*α*) and the acceleration of mortality with age (*β*). In contrast, while females continuously exposed to younger nonfocal females showed a similar increase in background mortality rate (*α*), the acceleration of mortality with age was smaller (long purple arrow points downward), which partially mitigated the longevity loss.

### Male Lifespan and Actuarial Aging Patterns

In contrast to the survival patterns observed for females, the variation in male lifespan was much less marked upon exposure to females or to other males, and there were no marked qualitative differences in the shape of lifespan decrease between any treatments (Figure 2B). Male survival was affected by exposure to nonfocal individuals (RMST_K-M_ ANOVA; *F*_3,16_ = 32.65, *p* < .001) and the largest effect was observed in individuals exposed continuously to the opposite sex. Male lifespan was much less affected by the social environment manipulations (Figure 2, Table 1). The difference in lifespan between alone versus continuous exposure to the opposite sex treatments was almost 4 times smaller in males (post hoc RMST_K-M_ contrast = 7.0 days, *t* = 8.93, *p* < .001) than it was in females (“alone” females versus continuous exposure to males, post hoc RMST_K-M_ contrast = 25.3 days, *t* = 15.04, *p* < .001). There were no marked qualitative differences in the shape of lifespan decrease between any treatments (Figure 2B) and, as in females, initial density differences between treatments did not appear to influence the survival patterns to any great extent. Overall, males exposed to females only intermittently (RMST_GSM_ = 28.6 days [27.7, 29.4]) had similar lifespans (post hoc RMST_K-M_ contrast *t* = 1.06, *p* = .31) to the focal “alone” treatment males (RMST_GSM_ = 29.1 days [28.2, 30.0]), suggesting negligible costs of mating. As with continuous exposure to females, continuous exposure to young nonfocal males significantly reduced male lifespan, but by a much smaller magnitude (“alone” focal males versus continuous exposure to nonfocal males, post hoc RMST_K-M_ contrast = 3.7 days, *t* = 4.68, *p* < .001). Comparing across treatments initiated at the same densities showed that males continually exposed to females showed slightly shorter lifespans than did males exposed to nonfocal males (post hoc RMST_K-M_ contrast = 3.3 days, *t* = 4.24, *p* < .001), supporting the idea that overall survival patterns were not confounded by initial density differences.

When plotted on the same axes, the data on the aging parameters for males showed much smaller between-treatment variation in comparison to females (Figure 3, Table 1). Nevertheless, male aging was still significantly affected by social environment (permutational MANOVA; *F*_3,16_ = 11.61, *p* < .001). All post hoc contrasts were significant after Benjamini–Hochberg correction (apart from alone focal males versus intermittent (opposite sex) males, *p* = .66). As in the female experiment, different aging effects were found depending on the identity of the nonfocal sex. With increasing exposure to females, we found that males experienced a sizeable increase in initial (background) rate of mortality (*α*) with a small drop in the acceleration of mortality with age (*β*). In contrast, males exposed to young nonfocal males showed no increase in background mortality rate (*α*), but a small increase in the acceleration of mortality parameter (*β*).

Comparing the survival patterns of males to females, we observed that, in single sex groups, males lived much shorter lives than females. However, under continuous exposure to young individuals of the opposite sex, males had a very similar lifespan to that of the corresponding females, reversing the difference in sexual dimorphism for lifespan (RMST_GSM_, continuously exposed males = 25.1 days [24.3–25.8], RMST_GSM_ continuously exposed females = 19.1 days [17.8–20.4]). This confirms that the impact of exposure to the opposite sex was much greater for females than for males (Figure 1A and B). This, together with the very different pattern of survival of focal alone (F) versus focal + nonfocal (FF) females, and the corresponding groups of M versus MM males, confirms different types of lifespan responses across the sexes.

## Discussion

Our main aim was to test the hypothesis that there are qualitatively and quantitatively different effects of same versus opposite sex exposure on mortality rates in males and females. The findings support this idea and provide new results showing opposing effects of same versus opposite sex exposure on survival and actuarial senescence in males and females. The results support existing research showing that the impact of each sex upon the survival of the other can be markedly different (14,15,28,29,31). For example, continual exposure to males reduced female lifespan by 50%, whereas continual exposure to females had little impact on male lifespan. We were also able to compare the effects of same sex exposure, which were also contrasting across males and females, with elevated same sex exposure in females causing a persistent loss of survival across the whole lifespan, yet very little impact of the same social environment in males. Increased exposure to the same or opposite sex increased the initial mortality rate in both sexes. However, increased exposure to the opposite sex markedly increased the rate of aging in females and decreased it slightly in males. In contrast, increased exposure to the same sex appeared to decrease the rate of aging in females, while slightly elevating it in males. These findings show that exposure to the same versus opposite sex led to differences in the rate of aging itself in opposing patterns in females versus males. This has potentially significant ramifications (83) and supports the idea that the nature and impact of same or opposite sex interactions are sex-specific, and can contribute to observed sex differences in lifespan. Therefore, studies that do not consider the impact of this sex-specificity may overlook important components of lifespan determination.

### Contribution of Opposite Sex Exposure to Sex Differences in Lifespan

The results indicate that any given interaction with the opposite sex is likely to have a much greater impact upon female survival, mortality rate, and potential fitness, than is true for males. This is consistent with a large body of previous research findings (14,15,28,29,38–51,64). In addition, the findings support Promislow’s prediction (15)—that sexual conflict between the sexes, arising from sex-specific costs, can be a significant predictor of sex differences in lifespan (84). Here, we used large cohorts to detect robust differences in aging rate parameters, as in Refs (64,85). Therefore, we have only a composite measure of the contribution of both pre- and postmating interactions of each sex to the survival patterns of the other. The relative importance of those effects could differ for each sex and may include the extent to which costs arise from “perception” versus actual energy allocation costs (34,86,87). These results are important because many experimental studies that seek to explain the genetic and environmental contributions to lifespan determination focus on nonreproductive or once-mated individuals separated from the other sex after early age. Such studies would overlook the impact on survival that is mediated by biotic interactions with the other sex (20,64,88) including those arising from sexual conflict (15), which themselves may also interact with other factors such as diet (89). The extent to which the impact of each sex upon survival and aging of the other is due to perception versus energy allocation decisions, and whether this also differs between the sexes, is also an interesting question for future studies (34,86,87).

The results in terms of the differences observed in aging parameters are potentially important. Studies that vary the environment of individuals by varying diets or same/opposite sex exposure and that deconvolute the patterns into aging parameters have often observed changes primarily to initial mortality rates rather than to the rate of aging itself (64,90,91). Here, we observed variation in both parameters, and in particular, the rate of aging in females increased upon elevated exposure to males, an effect that was not observed in males. Changes in both aging parameters have been observed in experiments in which the shape of the life history is selected, eg, upon selection for age at reproduction (91) and in response to environmental variation in temperature (92). Future analyses of these types of survival patterns using a pace-shape framework for aging (93) might be useful, as the results for the females do suggest the presence of underlying changes in the shape of aging.

### Contribution of Same Sex Exposure to Sex Differences in Lifespan

Continual exposure of females to young nonfocal individuals of the same sex induced qualitative and quantitative differences in survival in comparison to focal females held together without this exposure. There were again opposing effects in males. These findings fit within a growing body of research showing sex-specific effects of the sociosexual environment on lifespan (20,61,83,94,95), including studies that show effects on lifespan arising due to the age of the social partners with which focal individuals are kept (61,95). Here, we found that the impact of exposure to young individuals of the same sex throughout life caused a significant loss of lifespan in females but to a much lesser extent than was found for exposure to males (for both intermittent and continuous exposure to male treatments). In males, the effect of exposure to nonfocal younger males was much smaller and equivalent in terms of loss in length of life to the effect of exposure to the opposite sex, in line with previous studies in other species (31).

The survival trajectory in the same sex exposure treatments was different for each sex, and evident as a relatively “flat” survival curve in females exposed to young nonfocal females, but not in males exposed to nonfocal males. The flat curve in females suggests that there was a constant loss of mortality in females with time. Why females continuously exposed to nonfocal individuals of the same sex should experience a relatively constant probability of death throughout life is unclear. It could reflect a general level of continual extrinsic stress experienced by the focal females. However, food was provided ad libitum, and it seems unlikely that females were nutritionally stressed. Nor were any of these cages typified by high numbers of same sex interactions (in comparison to the frequent intersexual interactions observed in cages containing both sexes). Hence, it appears that some aspect of exposure to young individuals of the same sex had a deleterious impact upon female lifespan. The exact mechanisms involved will be interesting to probe further (83).

## Summary

Overall, our results contribute to understanding sex differences in lifespan and show the significant impacts of sex-specific reproductive costs on lifespan and actuarial senescence. They show the importance of considering the varying nature of social exposures and the constant expression of reproductive costs in each sex. We suggest that the results highlight important determinants of aging itself that need to be considered more widely. The results also contribute knowledge to a debate about actuarial aging (91). For example, it has been suggested that phenotypic manipulations of the external environment (environmental toxins (90), temperature (92)) can predominately impact the rate of aging, whereas phenotypic manipulations of the social and mating environment alter instead the baseline level of mortality (64). However, exactly how rigorous hypothesis is and whether it holds for both sexes, has not yet been resolved. Our findings here help, by showing that sociosexual exposure can affect both parameters of aging but differently in each sex, with changes to the rate of aging being a notable feature of survival effects in females. This fits with previous findings that survival costs of reproduction in males, but not females can be reversed (41,96), implying that a key factor for variation in male survival is instantaneous risk, and in females is aging rate.

## Supplementary Material

glad215_suppl_Supplementary_Tables_S1

## Data Deposition

Data are deposited in the Figshare data depository (10.6084/m9.figshare.24018867).
